# Cell Surface Parameters for Accessing Neutrophil Activation Level with Atomic Force Microscopy

**DOI:** 10.3390/cells13040306

**Published:** 2024-02-07

**Authors:** Oksana M. Tilinova, Vladimir Inozemtsev, Ekaterina Sherstyukova, Snezhanna Kandrashina, Mikhail Pisarev, Andrey Grechko, Nina Vorobjeva, Viktoria Sergunova, Maxim E. Dokukin

**Affiliations:** 1 Sarov Physics and Technology Institute, MEPhI, 607186 Sarov, Russia; oxsana.tilinova@gmail.com; 2 Federal Research and Clinical Center of Intensive Care Medicine and Rehabilitology, V.A. Negovsky Research Institute of General Reanimatology, 107031 Moscow, Russia; va.inozemcev@physics.msu.ru (V.I.); kmanchenko@yandex.ru (E.S.); snezhanna.lyapunova@yandex.ru (S.K.); pisarev@gmail.com (M.P.); avgrechko@fnkcrr.ru (A.G.); 3 Department of Immunology, Biology Faculty, Lomonosov Moscow State University, 119234 Moscow, Russia; nvvorobjeva@mail.ru

**Keywords:** atomic force microscopy, Ringing mode, neutrophil, activation, adhesion, glycocalyx, cell surface, reactive oxygen

## Abstract

In this study, we examine the topography and adhesion images of the cell surface of neutrophils during the activation process. Our analysis of cell surface parameters indicates that the most significant changes in neutrophils occur within the first 30 min of activation, suggesting that reactive oxygen species may require approximately this amount of time to activate the cells. Interestingly, we observed surface granular structure as early as 10 min after neutrophil activation when examining atomic force microscopy images. This finding aligns with the reorganization observed within the cells under confocal laser scanning microscopy. By analyzing the cell surface images of adhesion, we identified three spatial surface parameters that correlate with the activation time. This finding enables us to estimate the degree of activation by using atomic force microscopy maps of the cell surface.

## 1. Introduction

Atomic force microscopy (AFM) is a highly versatile technique for investigating the properties of complex structures and materials with exceptional spatial resolution [1,2,3]. Today, AFM is capable of analyzing the topographic, mechanical [1,2,4], chemical [5], electrical [6], and magnetic [7] properties of objects. Recently, there has been an increase in the use of combined measurement techniques that allow for the comprehensive analysis of complex objects such as nanocomposites, polymers, and biological systems [3,8]. Leading microscope manufacturers have introduced multimodal techniques that allow for the extraction of different physical parameters from a single experiment [9].

In recent years, AFM has found extensive application in the analysis of biological objects [10,11], especially cells [12,13,14]. There are many studies reporting the differences in the physical properties of cancer and healthy cells, as identified by AFM [15,16,17,18]. Most of them are based on mechanical properties of cells which depend on many factors and can be difficult to measure accurately [19,20].

AFM imaging provides the most accurate information about the physical properties of living cell surfaces. However, there are many limitations to working with such objects. For example, when working in a liquid environment, the advantage of obtaining high-resolution images of the cell surface is counteracted by the Brownian motion of the pericellular layer, which can also be altered by the AFM probe itself during scanning.

The way to overcome this problem is to use fixed cells and analyze the cell surface structure [21]. The pericellular layer, or glycocalyx, is a layer of polysaccharides and glycoproteins attached to the plasma membrane in many eukaryotic and Gram-negative prokaryotic cells. The detection of this layer can be achieved using the particle exclusion technique [22,23].

High-resolution maps of the cell surface can be obtained by fixing the cell, thereby creating a dense surface coat of the pericellular layer. This layer retains information about the physical structure of the glycocalyx prior to fixation. Mapping and analyzing the adhesion properties of the cell surface after fixation provide insight into both the cell surface and glycocalyx structure, which can be related to cell functionality and phenotype [24,25]. This approach was shown to help detect all stages of cancer progression in human cervical epithelial cells [26].

Although AFM imaging of fixed cells is capable of detecting human cervical cancer, it has been impossible to achieve such a precise separation between precancerous and cancerous cells. Using the recently introduced Ringing mode AFM modality, this problem can be solved by analyzing multiple cell surface parameters and machine learning data processing. AFM imaging of the adhesion maps of cells extracted from urine was shown to detect active bladder cancer with 94% accuracy [27]. In addition, this approach allows for the differentiation of cancer cells with different neoplastic behavior [28].

AFM can provide both topographic images and maps of surface physical properties with remarkable resolution, even down to the sub-nanometer scale [29]. This capability is particularly advantageous for obtaining complex images of cell surfaces, which often exhibit significant heterogeneity on a scale of up to 100 nm. Such images can be invaluable in addressing current challenges in biology and medicine. Moreover, the combination of AFM cell imaging with machine learning could be used to identify cell types at the single-cell level with relatively high precision.

Neutrophils, the most abundant type of immune cells in the human body, play crucial roles in responding to foreign agents and preventing their spread [30]. The glycocalyx in neutrophils not only provides structural integrity, but also plays an important role in immune responses. The membrane surface of neutrophils contains selectins, integrins, and other molecules that facilitate adhesion to endothelial cells during inflammatory responses [31,32,33]. Once attached to the endothelial surface, neutrophils migrate across the endothelium to sites of infection or inflammation [34]. The glycocalyx acts as a protective shield against external mechanical stress and potentially harmful agents in the bloodstream. Its degradation, as observed under certain conditions, may render neutrophils more susceptible to damage [35]. In addition, the neutrophil glycocalyx also modulates the cell’s respiratory burst, which involves the rapid release of reactive oxygen species that are necessary for antimicrobial effects [36,37,38].

It is important to note that stimulation of neutrophils, with both physiological and pharmacological stimuli, such as hydrogen peroxide [39,40,41], not only activates effector functions, but also leads to significant changes in the shape of the plasma membrane, including spreading [42]. The mechanisms behind this spreading phenomenon have been described previously [43] and are controlled by a calcium-dependent cysteine proteinase called calpain [43]. It has been suggested that, in their inactive state, neutrophils have a folded plasma membrane that acts as a ‘reservoir’ of the extra membrane needed for cell spreading [43,44]. These cell surface folds are held together by proteins, such as talin and ezrin, which link the plasma membrane to the underlying cortical actin network [45,46]. These proteins are known substrates of calpain-1 [43]. The activity of calpain-1 is highly dependent on an increase in the cytoplasmic calcium concentration, which activates the enzyme and degrades proteins that support the folds. Therefore, when neutrophils are activated, both the glycocalyx and the plasma membrane undergo significant structural changes.

Despite ongoing research, many questions about neutrophil structure and function remain unanswered. Modern optical methods, in particular, do not fully reveal the mechanisms of functioning of these cells, or the processes occurring within them [47]. Therefore, there is an urgent need for new research techniques that will improve our understanding of this critical component of the immune system. This study aimed to evaluate neutrophil surface changes during activation by reactive oxygen species, which can serve as a foundation for a more comprehensive analysis of the biophysical processes of activation.

## 2. Materials and Methods

### 2.1. Cells

Neutrophils isolated from the blood of healthy donors were used in this study. All experiments involving blood were conducted in compliance with the Helsinki Declaration on Ethical Principles for Medical Research (2000) and the Protocol to the Convention for the Protection of Human Rights and Dignity of the Human Being with regard to the Application of Biology and Medicine (1999).

To isolate neutrophils, fresh donor blood was layered on a Ficoll double gradient (Paneco, Moscow, Russian Federation) (d_1_ = 1.119 g/cm^3^, d_2_ = 1.077 g/cm^3^) and centrifuged at 400× *g* for 40 min at room temperature. The layer containing neutrophils with erythrocyte admixture was then selected. The collected sample was lysed in hypotonic saline (0.2%) at 4 °C for 30 s, followed by 2× phosphate-buffered saline (PBS) to remove the erythrocytes. The isolated neutrophils were washed in 1× PBS solution before being resuspended in RPMI 1640 (Paneco, Russian Federation) supplemented with 10 mM HEPES (Paneco, Russian Federation), 2 mM L-glutamine (Paneco, Russian Federation), and 1% heat-inactivated fetal calf serum (Paneco, Russian Federation). Visual microscopy of the isolated cells showed that neutrophils constituted more than 97% of the total number of cells. The trypan blue viability of the cells was more than 95%. Prior to use in the experiment, neutrophils were stored at 4 °C for no longer than 30 min.

To adhere the cells to the glass, freshly isolated neutrophils (2 × 10^5^ cells/mL) were placed on sterile uncoated round coverslips and incubated at 37 °C and 5% CO_2_ for 30 min. To activate the neutrophils, 2 mM hydrogen peroxide (H_2_O_2_) was added.

After incubation with H_2_O_2_, samples were fixed with 4% paraformaldehyde for 30 min at room temperature and washed three times in 1× PBS. To remove salts, cells were rinsed in distilled water for 30 s. All samples were rapidly washed twice with distilled water and dried in a vacuum desiccator (Jeio Tech VDC-11U, Daejeon, Republic of Korea) at 1 × 10^−3^ MPa for 1 h.

### 2.2. Confocal Microscopy

For confocal microscopy, neutrophils were treated with 0.05% Triton X-100 (Sigma, USA) in 1× PBS for 15 min at 4 °C. The neutrophils were then rinsed with 1× PBS and blocked with 3% bovine serum albumin (BSA) (Paneco, Russian Federation) to prevent non-specific binding. DNA was stained using Hoechst 33342 dye (Sigma, St. Louis, MO, USA), which was diluted 1:1000 in 1× PBS and applied for 20 min. For plasma membrane staining, Alexa Fluor 594-conjugated Wheat Germ Agglutinin (WGA) (Thermo Fisher Scientific, Waltham, MA, USA) was used at 1:500 dilution in 1× PBS for 30 min. Throughout the staining process, the samples were protected from light. After staining, the coverslips were washed three times with 1× PBS and mounted on glass slides using Abberior mount solid antifade (Abberior, Göttingen, Germany).

The 405 nm laser wavelength was used to excite the Hoechst 33342 dye, and the 543 nm laser wavelength was used for Alexa Fluor 594 WGA. All images were captured using a Zeiss LSM880 confocal laser scanning microscope (CLSM) (Carl Zeiss, Jena, Germany) with the Airyscan module and a 63× immersion objective. Z-stacks of 10 individual cells were collected for each activation time. ImageJ 1.54f software [48] was used for all image preprocessing, including the transformation of the acquired z-stacks.

### 2.3. Atomic Force Microscopy

The NTEGRA Prima atomic force microscope (NT-MDT Spectrum Instruments, Moscow, Zelenograd, Russia) was used in this study. The Ringing mode technique [29], which is based on the analysis of the free oscillatory motion of the AFM cantilever that occurs after the probe is disconnected from the sample surface, was used to capture restored and viscoelastic adhesion maps. All measurements were performed using HybriD sub-resonance mode (NT-MDT Spectrum Instruments, Moscow, Zelenograd, Russia). The SPIP 6.5 software (Image Metrology A/S, Lyngby, Denmark) was used for offline data processing.

Prior to each measurement, the cantilever was fully calibrated, including the vertical deflection sensitivity and the spring constant. The deflection sensitivity was calibrated against a clean glass slide. The Sader method (power spectral density analysis of thermally driven cantilever oscillations), integrated into the HybriD mode, was used to calibrate the cantilever spring constant.

To acquire images of the cell surface, SCANASYST-AIR probes (Bruker, Santa Barbara, CA, USA) with a nominal spring constant of 0.4 N/m and a resonance frequency of 70–80 kHz were used. The probe radius was ~3 nm.

For each sample type, up to 20 cell images were acquired with a resolution of 256 × 256 pixels and a size of 5 × 5 μm^2^. The evaluation images used for the initial cell surface assessment were 15 × 15 μm^2^ in size and had a resolution of 256 × 256 or 512 × 512 pixels. The maximum loading force applied to the sample during scanning was 2 nN. The scanning speed of 0.3 Hz was chosen for all images, as a compromise between speed and image quality. The optical system integrated into the AFM was used to position the probe during the experiment.

### 2.4. Ringing Mode

The Ringing mode was modified to work with the HybriD mode technique. In this work, in addition to the 6 data channels (topography, adhesion, stiffness, elastic modulus, deformation, and viscoelastic energy loss) available in HybriD mode, restored and viscoelastic adhesion maps were collected using Ringing mode. Further details of the Ringing mode are discussed in [28,29]. The technique is summarized below.

**Figure 1:**
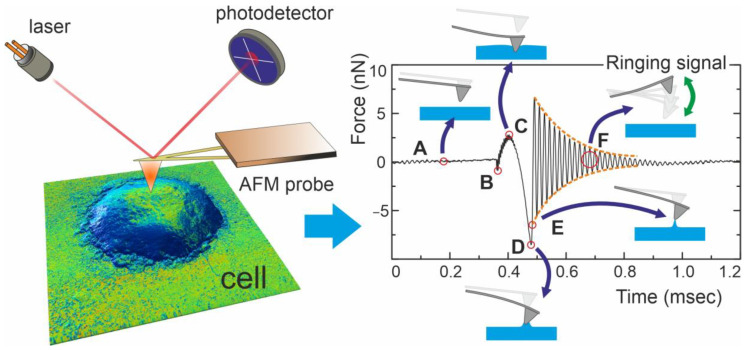
Schematic representation of Ringing mode data acquisition. Cell surface images were acquired in HybriD mode (**left**). A complete cycle of probe interaction with a cell surface is shown (**right**). Specific points during the probe–sample interaction: (A) before contact, (B) initial contact with the sample, (C) deformation of the sample surface at the maximum load force, (D) start of rapid detachment from the sample (the adhesion force), (E) complete detachment from the sample (the restored adhesion force), and (F) free oscillations above the sample surface (ringing signal).

Figure 1 shows a schematic example of Ringing mode imaging. After detachment from the surface, the cantilever continues to oscillate at its resonant frequency (~70–80 kHz) (region F, Figure 1). Considering this motion of the cantilever as the motion of a damped free oscillator, we can recover the value of the force acting on the cantilever at the moment of complete detachment from the surface (point E, Figure 1). This parameter characterizes the restored adhesion.

Another parameter characterizing the interaction between the probe and the surface is viscoelastic adhesion, which is calculated as the difference between the adhesion force (point D, Figure 1) and the restored adhesion force (point E, Figure 1). The physical meaning of this parameter is the change in the force exerted by the sample on the cantilever, due to the loss of energy caused by the inelastic deformation of the sample during the probe detachment. When working with soft materials, such as fixed cells, these two types of adhesion show maximum sensitivity to the local geometry and physical properties of the cell surface. It is worth noting that adhesion measurement allows for obtaining cell surface maps with a resolution down to 1 nm [29], which makes these images optimal for evaluating changes in the surface properties of neutrophils during their activation.

### 2.5. Cell Surface Parameters

Surface parameters are commonly used in engineering applications for the description and categorization of surfaces. These parameters can also be used to study surface changes in biological objects, such as cells [27].

In this work, cell surface parameters were calculated according to specific standards (ISO 4287/1 ASME B46.1 [49] and ISO/DIS 25178-2) [50] using SPIP 6.5 software (Image Metrology A/S, Lyngby, Denmark). All calculations were performed for height images and maps of restored and viscoelastic adhesion. In the case of adhesion, the calculation was performed for the so-called effective height, which corresponds to the value of adhesion at each image point. Surface parameters were calculated for images measuring 5 × 5 μm^2^ with a resolution of 20 nm/pixel. For each type of data, 37 surface parameters were calculated [27].

To estimate surface parameters, all collected AFM images were used as is, except the height maps. This exclusion is because the absolute value of the height on the AFM maps does not make physical sense, since the origin of the coordinates may be different for each experiment. Therefore, to calculate the parameters of the height maps, each height image was pre-normalized by removing the plane tilt in the SPIP software.

It should be noted that, before calculating the surface parameters, each image was visually examined for scanning artifacts and/or foreign objects on the cell surface. Images containing such artifacts or objects were excluded to obtain a more precise analysis of the changes in the cell surface.

## 3. Results and Discussion

### 3.1. Confocal Images

**Figure 2:**
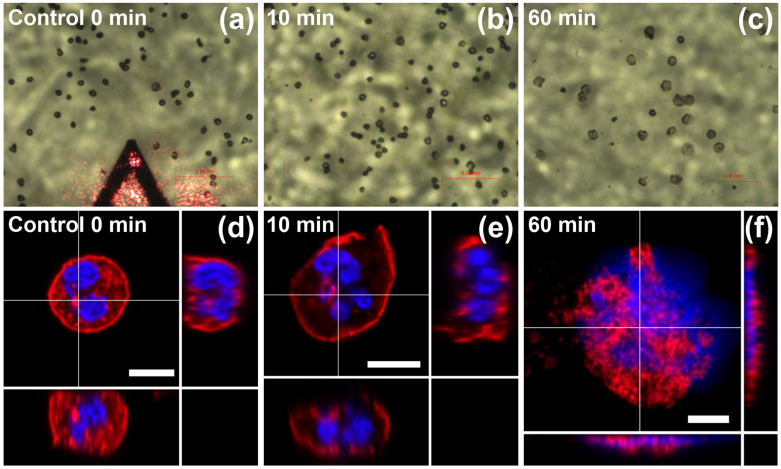
Representative images of cells with bright-field AFM embedded optics (**a**–**c**). The scale bar shown on all bright field images is 80 μm. The black formations in figures (**a**–**c**) correspond to neutrophil cells. As can be seen, the lateral size of the cells increases with the activation time. Confocal cell images with cross sections are shown in (**d**–**f**). The neutrophil membrane is stained with WGA + AlexaFluor 594 (red), and the nucleus is stained with Hoechst33342 (blue). All confocal scale bars are 5 μm. Control samples, before activation (0 min) and incubated in the medium with added H_2_O_2_ for 10 and 60 min, are shown. The crosshairs correspond to the positions of the vertical and horizontal sections. The AFM cantilever (in (**a**)) is shown for scale.

Prior to the AFM analysis, neutrophils were evaluated using CLSM and integrated AFM optics (Figure 2). Neutrophils in the control group were mostly spherical or hemispherical cells with segmented nuclei (Figure 2d). These cells appear dark in bright-field images (Figure 2a). At 5–10 min after the addition of hydrogen peroxide, the shape of the neutrophil became irregular. However, the membrane retained its integrity and the nucleus was still segmented (Figure 2e). The center of the cell appeared brighter in bright-field microscopy (Figure 2b), indicating a decrease in cell thickness. After approximately 30 min in a hydrogen peroxide medium, the cell membrane started to degrade with characteristic granularity, but the nucleus remained segmented. After 60 min, the cell was completely degraded; the nucleus lost its segmented structure and was destroyed (the cell became flat) (Figure 2c,f). No further significant changes in neutrophil structure were observed after 60 min.

### 3.2. Visualization of Cell Surface Adhesion Maps

**Figure 3:**
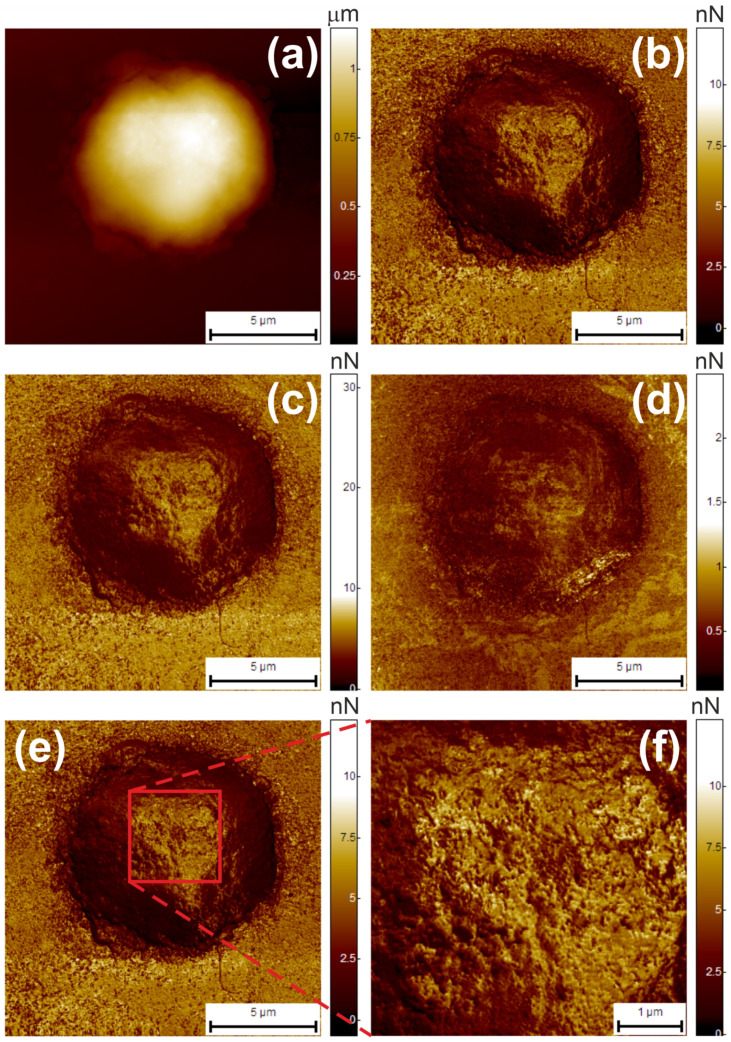
Typical AFM images of the neutrophil surface. Maps of height (topography) (**a**), restored adhesion (**b**), viscoelastic adhesion (**c**), and regular adhesion (**d**) are shown. All images (**a**–**d**) were acquired simultaneously. The control cell was used. An example of area selection within a cell is shown in (**e**,**f**). Low-resolution (60 nm/pixel) image of a neutrophil with the surrounding substrate (**e**) and the selected area (20 nm/pixel) used for parameter calculation (**f**).

To collect cell surface adhesion maps, the AFM probe was positioned over the cell using an integrated AFM optical system. This procedure is illustrated in Figure 2a. To evaluate its position over the cell, a low-resolution (up to 60 nm/pixel) test scan was performed. An example of the resulting image is shown in Figure 3. The figure shows maps of height (topography) (Figure 3a), restored adhesion (Figure 3b), viscoelastic adhesion (Figure 3c), and adhesion collected using the HybriD mode technique (Figure 3d).

It is evident that the adhesion maps obtained using the Ringing mode technique have significantly higher quality compared to the adhesion images acquired using the HybriD mode.

The reason for this difference is that, in HybriD mode, each pixel of the adhesion image is represented by only one data point, which corresponds to the minimum force. However, this limitation prevents an accurate determination of the parameter, due to the noise present in the measured signal. On the other hand, the Ringing mode technique calculates each value of the restored adhesion, based on the movement of the AFM cantilever after the probe is detached from the cell surface. This approach allows for a larger number of points to be used in the calculation, effectively eliminating the influence of noise and external factors, such as interference from the reflected laser beam. A detailed explanation of this technique can be found in reference [29].

After obtaining a preliminary image, the microscope software was used to select the central region of the cell so that the 5 × 5 µm^2^ area required for scanning was entirely located within the cell. An example of the selection is shown in Figure 3e,f. Figure 3e shows an adhesion map of a neutrophil surface and its surrounding substrate at low resolution. Figure 3f is the central area of the cell, scanned at high resolution (20 nm/pixel).

**Figure 4:**
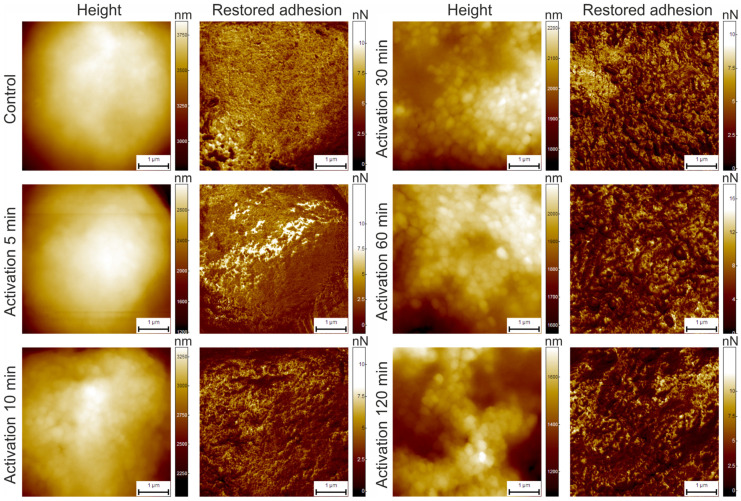
Typical height and restored adhesion AFM maps. Cell surface for the control sample (after 120 min in a culture medium) and samples 5, 10, 30, 60, and 120 min after activation are shown. All images are 5 × 5 μm^2^ (256 × 256 pixels). Images of height and restored adhesion for each cell were acquired simultaneously, using HybriD mode (height) and Ringing mode (restored adhesion).

Characteristic examples of AFM maps for neutrophils with different activation times are presented in Figure 4. The figure shows images of height (1st and 3rd column) and restored adhesion (2nd and 4th column). All images were acquired at the resolution of 256 × 256 pixels and were 5 × 5 µm^2^ in size. Each pixel in these maps has a defined numerical value (higher brightness means higher value of either height or adhesion). It can be seen that control cells, after 120 min incubation in the culture medium without H_2_O_2_, look smoother in the restored adhesion channel compared to activated cells.

The images of control cells before treatment (0 min) are omitted because they are almost identical to the control cells after 120 min in the medium without H_2_O_2_.

It can be seen in Figure 4 that, after 10 min of activation in a medium with added H_2_O_2_, granular formations become visible on the cell surface. Subsequent increases in activation time result in a more pronounced granular surface structure, and these changes are visually more prominent on the restored adhesion maps.

For each sample type, including two control groups, up to 20 cells were imaged. Each cell map consisted of 8 data channels, namely, height, adhesion, adhesion loss, deformation, stiffness, and elastic modulus (channels are available in HybriD mode), as well as additional channels of restored and viscoelastic adhesion (channels are available in Ringing mode). As noted above, the Ringing mode data channels have better image quality compared to the HybriD mode channels (see Figure 3). Therefore, only the height (HybriD mode), restored adhesion, and viscoelastic adhesion (Ringing mode) images were used for all subsequent calculations in this study.

### 3.3. Analysis of the AFM Images

**Figure 5:**
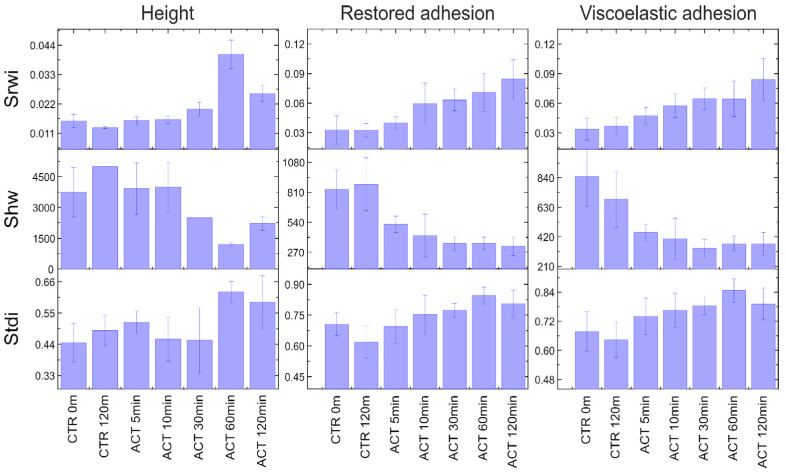
Values of S_rwi_, S_hw_, and S_tdi_ parameters versus the activation time (ACT 5–120 min). Data are shown for the height (**left column**), restored adhesion (**middle column**), and viscoelastic adhesion (**right column**) channels. The first two values in each graph are for control samples before activation (CTR 0m) and after 120 min in the culture medium without H_2_O_2_ (CTR 120 m).

Thirty-seven surface parameters were calculated for each type of AFM map. Of all the calculated parameters, only three were found to correlate with the activation time. Figure 5 shows the values of the S_rwi_, S_hw_, and S_tdi_ parameters as functions of activation time. One can see that the S_rwi_ and S_hw_ parameters for adhesion are dependent on the activation time. The values for control cells before activation and after 120 min in a culture medium without H_2_O_2_ remain practically unchanged.

The parameters S_rwi_, S_hw_, and S_tdi_ belong to the group of spatial parameters responsible for determining the surface anisotropy and periodicity. The calculation of the spatial parameters is based on the use of the 2D Fourier transform of sample surface maps.

The S_tdi_ parameter is a texture direction index. This parameter is calculated using a 2D Fourier spectrum and shows the magnitude of the dominant direction with respect to all other directions. To estimate this parameter, the relative magnitudes for the different angles (directions) on the 2D Fourier spectrum map are calculated by summation of the amplitudes along M equiangularly spaced radial lines. The result is called the angular spectrum. Notably, the Fourier spectrum is translated so that the DC component is in the center of the 2D Fourier map. The angle of the *i*-th line is equal to π/M, where M is the image size in pixels (for the Fourier transform, we assume that the image is quadrangular). S_tdi_ is the ratio of the average amplitude to the total amplitude of the dominant direction. Its value varies between 0 and 1. The greater the degree of dominance, the closer its value is to 0. If there is no dominant direction, this parameter tends toward 1.

The S_rwi_ is the radial wave index. It characterizes the spatial structure of the sample surface and shows the magnitude of the dominant radial wavelength. The dominant wavelength was found in the radial spectrum of the 2D Fourier map. The radial spectrum is calculated by summation of the amplitude values around M/(2–1) equally spaced semicircles, with the center at the center of the 2D Fourier map. The radii of the semicircles are measured in pixels, and r is in the range of 1, 2, …, M/(2–1).

S_rwi_ is the ratio of the average amplitude to the total amplitude of the dominant wavelength. S_rwi_ also varies between 0 and 1. If the dominant wavelength is present, this parameter is close to 0. If there is no dominant wavelength, this parameter is close to 1.

S_hw_ is the average half wavelength and also characterizes the average spatial structure of the surface, using the concept of the radial spectrum. S_hw_ corresponds to the radius $r_{0.5}$, which is obtained as follows: if $\beta_{i}\left(r\right)=\sum_{j=1}^{r}\beta (j)$ is the integral radial spectrum, then $\frac{\beta_{i}(r_{0.5})}{\beta_{i}(M/2–1)}=0.5$ and $S_{hw}=\frac{\delta x(M–1)}{r_{0.5}}$.

All of these parameters are explained in detail in the SPIP manual [50].

The above parameters, S_rwi_, S_hw_, and S_tdi_, were also calculated for the height images (Figure 5, first column). Unlike the data for the restored and viscoelastic adhesion channels, those for the height channel do not show any correlation between these parameters and the activation time. As previously discussed, this may be due to the fact that adhesion parameters are more sensitive to local changes in both geometry and the physical/compositional properties of the cell surface [29].

Although S_rwi_ and S_hw_ vary with time, for both restored and viscoelastic adhesion channels, these parameters can only be used to evaluate the level of neutrophil activation for the restored adhesion channel.

### 3.4. Effect of the Activation Process on Surface Parameters

During activation, neutrophils can significantly change their morphology and, in particular, their shape, e.g., from spherical to flat (spread) [42,51]. This process may also be accompanied by changes in the pericellular layer (glycocalyx), which are difficult to evaluate using only classical optical methods. Meanwhile, the use of AFM makes it possible to obtain detailed images of the neutrophil cell surface, which allows for the development of criteria for estimating changes in cell surface at different levels of activation.

Analysis of the cell surface maps revealed a decrease in the S_hw_ parameter for channels of restored and viscoelastic adhesion with increasing activation time. This indicates that the membrane becomes rougher with a prominent granular structure. Since the lateral size of the cell increases significantly during activation, the granular structures detected by the AFM on the cell surface could be the manifestation of cytoplasmic granules. This is also supported by confocal images of neutrophils taken within 30–60 min after activation (see Figure 2c,f).

The increases in S_rwi_ and S_tdi_ parameters also suggest a greater heterogeneity of the cell surface after activation. It was found that AFM enables the detection of significant alterations on the cell surface shortly after activation (less than 10 min), whereas optical methods fail to reveal any changes in the neutrophil membrane during this time.

**Figure 6:**
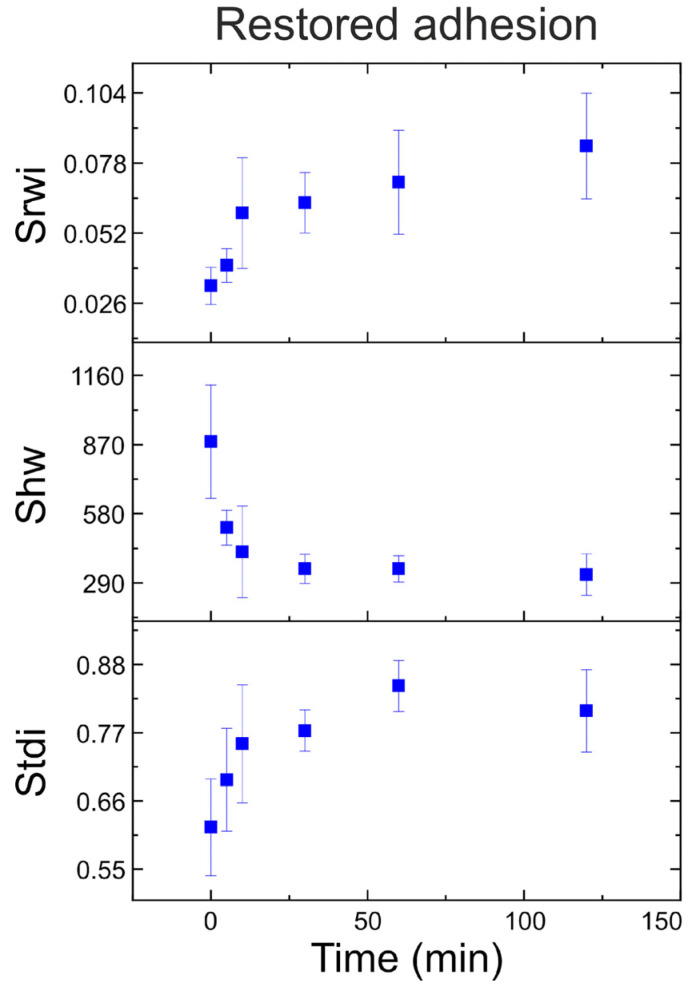
The dependence of each of the parameters, S_rwi_, S_hw_, and S_tdi_, on the activation time for the restored adhesion channel is shown.

Analysis of the time dependence of S_rwi_, S_hw_, and S_tdi_ shows that neutrophil activation is usually completed within the initial 30 min, and the cell surface remains virtually unchanged for the next 90 min in a medium containing reactive oxygen species. As can be seen in Figure 6, the parameters S_rwi_ and S_hw_ help to determine the level of activation, while S_tdi_ has lower values at 120 min compared to 60 min.

Thus, the AFM data can be correlated with the changes in the plasma membrane and the pericellular layer of cells and used to assess the level of neutrophil activation.

## 4. Conclusions

One of the key characteristics of neutrophils is their ability to change shape quickly. Neutrophils circulate in the bloodstream in an almost spherical shape, but are able to rapidly deform. This ability is an important feature that allows neutrophils to reach the area of tissue where they are needed in a short period of time.

Here, we analyzed the topography and adhesion images of the cell surface of neutrophils during the activation process. Significant changes in the cells’ surface structure were observed if neutrophils were activated in a medium containing reactive oxygen species (H_2_O_2_). Meanwhile, storage of neutrophils in a culture medium for 120 min (maximum activation time) does not result in any noticeable changes in the cell surface.

Analysis of the surface parameters revealed that the main changes in the neutrophils occur during the first 30 min of the activation process, which could indicate the approximate time required for the activation by reactive oxygen.

The specific response of neutrophil membranes to activation is manifested by changes in their structure. In areas where the membrane is stretched, a decrease in local surface physical adhesion of the membrane is observed (Figure 4). This decrease in membrane surface adhesion (expressed as the force required to detach the probe from the surface) and cell spreading reveals granules that are located directly beneath the membrane. This process can be compared to the stretching of a membrane that covers the granules like a “wet blanket”, providing direct contact between the membrane and the granules. However, in areas where the membrane retains its original structure with numerous folds, the surface physical adhesion of the membrane is significantly higher. This may indicate that these regions of the membrane are not exposed to the same reactive oxygen species or other activating factors, which, in turn, may have implications for the functional activity of neutrophils.

The Ringing mode adhesion data were found to provide the most comprehensive information on the neutrophil surface. As early as 10 min into the neutrophil activation process, the surface granular structure can be observed when analyzing the AFM images. This correlates with the confocal laser scanning microscopy data of restructuring inside the cells. Thus, during neutrophil activation, they lose regular spatial structure, resulting in the emergence of visible granular formation on the surface.

Evaluating the surface properties of neutrophils, we identified three spatial surface parameters, S_rwi_, S_hw_, and S_tdi_, that correlate with the activation time and allow us to estimate the level of activation while analyzing adhesion maps of the cell surface.

Importantly, this correlation can only be obtained using the Ringing mode of atomic force microscopy. This method is unique for use in biology and opens up new horizons for further research into nanoscale cellular changes in cell biology and medicine.

## Data Availability

The datasets used and analyzed during the current study are available from the corresponding authors upon request.
